# National Patterns of Outpatient Follow-Up Visits After Emergency Care for Acute Bronchiolitis

**DOI:** 10.1001/jamanetworkopen.2023.40082

**Published:** 2023-10-27

**Authors:** Daniel J. Shapiro, Florence T. Bourgeois, Andrew M. Fine, Adam L. Hersh, Eric R. Coon, Mark I. Neuman, Ann Chen Wu

**Affiliations:** 1 Division of Pediatric Emergency Medicine, University of California, San Francisco; 2 Computational Health Informatics Program, Boston Children’s Hospital, Boston, Massachusetts; 3 Division of Pediatric Emergency Medicine, Boston Children’s Hospital, Boston, Massachusetts; 4 Division of Pediatric Infectious Diseases, University of Utah, Salt Lake City; 5 Department of Pediatrics, Primary Children’s Hospital, University of Utah School of Medicine, Salt Lake City; 6 Department of Population Medicine, Harvard Medical School and Harvard Pilgrim Health Care Institute, Boston, Massachusetts

## Abstract

This cohort study examines the frequency of postdischarge follow-up visits among US emergency department encounters for bronchiolitis and assesses whether follow-up was associated with decreased hospital reutilization or increased treatment with nonrecommended medications.

## Introduction

Although the value of routine posthospitalization follow-up visits for otherwise healthy children with acute bronchiolitis has been questioned,^1^ less is known about the value of follow-up for children discharged from the emergency department (ED). Follow-up visits offer an opportunity to reassess the clinical trajectory and to provide reassurance to families, but they also may result in financial costs, missed work, and overtreatment.^2^ Although anecdotal evidence suggests that recommending postdischarge follow-up is the default practice for many ED clinicians, to our knowledge, national practice patterns have not been described. Our objectives were to describe the frequency of postdischarge follow-up visits in a national cohort of ED encounters for bronchiolitis and to assess whether follow-up was associated with decreased hospital reutilization or increased treatment with nonrecommended medications.

## Methods

This was a retrospective cohort study of ED discharges between 2018 and 2021 among children with commercial insurance aged less than 2 years with acute bronchiolitis in the MarketScan Commercial Claims and Encounters Database.^3^ The database includes insurance claims for more than 30 million employees and their dependents in the United States. We excluded children with complex chronic conditions.^4^ The Harvard Pilgrim Health Care Institute institutional review board approved this study. The need for informed consent was waived because data were obtained in a deidentified format. We followed the STROBE reporting guideline.

The primary outcome was a follow-up visit in primary care within 7 days of ED discharge. The secondary outcomes were ED revisits without hospitalization, hospitalization, and dispensation of medications not recommended by national guidelines (albuterol, corticosteroids, antibiotics).^5^ Administrative codes are listed in the eAppendix in Supplement 1.

We used Poisson regression with robust clustered SEs to identify characteristics associated with attending follow-up. We clustered on individuals to account for nonindependence of observations among children with more than 1 illness episode. To assess the association between follow-up and subsequent hospital reutilization, we used multivariable Cox proportional hazard models. These models included follow-up as a time-varying exposure and adjusted for year, age, sex, region, weekend discharge, number of outpatient and ED visits in the prior year, whether there was an outpatient visit for bronchiolitis in the 7 days prior to the index ED visit, and testing (viral testing, chest radiography) and treatments (albuterol, corticosteroids, antibiotics) at the index visit. Statistical significance was considered at a 2-sided *P* < .05. Analyses were performed using R version 3.2.1 (R Project for Statistical Computing) from December 2022 to July 2023.

## Results

Among 28 338 ED discharges (27 000 unique patients), 63.3% (95% CI, 62.8%-63.9%) were less than 1 year of age and 58.8% (95% CI, 58.2%-59.3%) were male. Follow-up within 7 days occurred for 47.6% (95% CI, 47.0%-48.1%) of discharges. The proportion of discharges with follow-up decreased from 48.9% (95% CI, 47.9%-49.9%) in 2018 to 43.4% (95% CI, 42.2%-44.5%) in 2021 (*P* < .001). Several demographic and clinical characteristics were associated with follow-up (Table 1).

**Table 1.** Characteristics of the Cohort and Attending a Follow-Up Visit (N = 28 338)

Characteristic	Proportion of discharges, % (95% CI)		Follow-up visit, adjusted risk ratio (95% CI)^a^
	Without follow-up visit (n = 14 862)	With follow-up visit (n = 13 476)	
Year			
2018	30.3 (29.5-31.0)	31.9 (31.1-32.7)	1 [Reference]
2019	30.2 (29.5-31.0)	33.6 (32.8-34.4)	0.96 (0.93-0.98)
2020	13.1 (12.6-13.7)	12.2 (11.6-12.7)	0.88 (0.85-0.92)
2021	26.4 (25.7-27.1)	22.3 (21.6-23.0)	0.85 (0.82-0.88)
Age >1 y	40.1 (39.4-40.9)	32.8 (32.0-33.6)	0.83 (0.81-0.85)
Sex			
Male	58.7 (57.9-59.5)	58.8 (58.0-59.7)	1.00 (0.97 to 1.02)
Female	41.3 (40.5-42.1)	41.2 (40.3-42.0)	1 [Reference]
United States Census region			
Northeast	12.4 (11.8-12.9)	15.4 (14.8-16.0)	1 [Reference]
North central	28.8 (28.1-29.5)	26.0 (25.2-26.7)	0.87 (0.84-0.90)
South	44.9 (44.1-45.7)	43.5 (42.7-44.3)	0.89 (0.86-0.92)
West	13.9 (13.4-14.5)	15.1 (14.5-15.8)	0.98 (0.95-1.03)
Weekend discharge	30.4 (29.7-31.2)	33.4 (32.6-34.2)	1.07 (1.05-1.10)
Outpatient visit for bronchiolitis within 7 d prior to the index visit	25.0 (24.3-25.7)	31.0 (30.2-31.8)	1.12 (1.09-1.15)
No. of outpatient visits in the prior year, median (IQR)	5 (2-8)	5 (3-9)	1.03 (1.02-1.03)
Any emergency department visit in the prior year	32.1 (31.4-32.9)	28.8 (28.0-29.6)	0.90 (0.87-0.92)
Testing and treatment at the index visit			
Viral testing	41.1 (40.4-41.9)	43.9 (43.0-44.7)	1.08 (1.05-1.10)
Chest radiography	40.0 (39.2-40.8)	41.1 (40.3-42.0)	1.02 (0.99-1.05)
Albuterol	21.6 (21.0-22.3)	24.5 (23.8-25.3)	1.07 (1.04-1.11)
Corticosteroids	9.5 (9.0-9.9)	10.1 (9.6-10.6)	1.02 (0.98-1.06)
Intravenous fluids	3.6 (3.3-3.9)	5.1 (4.8-5.5)	1.15 (1.10-1.21)

^a^ Adjusted risk ratios are from a multivariable Poisson regression model with robust clustered standard errors. Clustering was performed at the level of the patient.

Within 7 days after discharge, 17.6% (95% CI, 17.1%-18.0%) of discharges had an ED revisit and 5.2% (95% CI, 5.0%-5.5%) were subsequently hospitalized. Follow-up was not associated with ED revisits (adjusted hazard ratio [HR], 1.09; 95% CI, 0.93-1.27) but was positively associated with subsequent hospitalization (adjusted HR, 1.41; 95% CI, 1.20-1.65). Prescriptions for albuterol, corticosteroids, and antibiotics were filled more frequently among children who attended follow-up (Table 2). Among children who attended follow-up, 12.2% (95% CI, 11.5%-12.8%) filled a prescription for albuterol, 5.4% (95% CI, 4.9%-5.9%) for corticosteroids, and 18.5% (95% CI, 17.8%-19.3%) for antibiotics after the follow-up visit.

**Table 2.** Medications Dispensed After Discharge, Stratified by Follow-Up Status

Medication	Proportion with medication dispensation, % (95% CI)		*P* value^a^
	No follow-up	Follow-up	
Albuterol	15.0 (14.4-15.7)	26.6 (25.8-27.5)	<.001
Corticosteroids	9.9 (9.4-10.5)	13.4 (12.7-14.1)	<.001
Antibiotics	16.3 (15.6-17.0)	30.4 (29.5-31.4)	<.001

^a^ *P* values are from χ^2^ test.

## Discussion

Approximately half of commercially insured children with acute bronchiolitis completed a follow-up visit within 7 days of ED discharge. Follow-up visits were not associated with ED revisits. The association between follow-up and hospitalization could be explained by confounding by indication (ie, children with more severe illness may be more likely both to follow up and to be hospitalized) or by overdiagnosis at follow-up.^6^ Nonrecommended medications were dispensed for a modest proportion of children after follow-up. Limitations of this study included incomplete data to adjust for clinical severity, an inability to distinguish scheduled from unscheduled follow-up visits, and inclusion of only patients with commercial insurance. Given that follow-up visits are common, vary in frequency according to visit characteristics, have uncertain benefits, may result in overtreatment, and carry costs to families and the health care system, prospective investigation is warranted to determine which children with bronchiolitis may benefit from routine follow-up.

## Notes

Supplement 1. **eAppendix.** Administrative Codes

Supplement 2.
Data Sharing Statement

## References

[1] Coon ER, Destino LA, Greene TH, Vukin E, Stoddard G, Schroeder AR. Comparison of as-needed and scheduled posthospitalization follow-up for children hospitalized for bronchiolitis: the Bronchiolitis Follow-up Intervention Trial (BeneFIT) randomized clinical trial. JAMA Pediatr. 2020;174(9):e201937. doi:10.1001/jamapediatrics.2020.1937 32628250 PMC7489830

[2] Coon ER, Conroy MB, Ray KN. Posthospitalization follow-up: always needed or as needed? Hosp Pediatr. 2021;11(10):e270-e273. doi:10.1542/hpeds.2021-005880 34479947

[3] IBM. IBM MarketScan Research Databases. Accessed August 2, 2022. https://www.ibm.com/products/marketscan-research-databases

[4] Feudtner C, Feinstein JA, Zhong W, Hall M, Dai D. Pediatric complex chronic conditions classification system version 2: updated for ICD-10 and complex medical technology dependence and transplantation. BMC Pediatr. 2014;14:199. doi:10.1186/1471-2431-14-199 25102958 PMC4134331

[5] Ralston SL, Lieberthal AS, Meissner HC, ; American Academy of Pediatrics. Clinical practice guideline: the diagnosis, management, and prevention of bronchiolitis. Pediatrics. 2014;134(5):e1474-e1502. doi:10.1542/peds.2014-2742 25349312

[6] Auger KA, Simmons JM, Tubbs-Cooley HL, ; H2O Trial study group. Postdischarge Nurse Home Visits and Reuse: The Hospital to Home Outcomes (H2O) Trial. Pediatrics. 2018;142(1):e20173919. doi:10.1542/peds.2017-3919 29934295 PMC12747583

