# Supplementary material for: National Patterns of Outpatient Follow-Up Visits After Emergency Care for Acute Bronchiolitis
Source: JAMA Netw Open. 2023 Oct 27;6(10):e2340082. doi: 10.1001/jamanetworkopen.2023.40082 (PMC10611989; doi:10.1001/jamanetworkopen.2023.40082)
Supplement: Supplement 1. — eAppendix. Administrative Codes [file jamanetwopen-e2340082-s001.pdf]

Supplemental Online Content

Shapiro DJ, Bourgeois FT, Fine AM, et al. National patterns of outpatient follow-up visits after emergency care for acute bronchiolitis. *JAMA Netw Open.* 2023;6(10):e2340082. doi:10.1001/jamanetworkopen.2023.40082

eAppendix. Administrative Codes

This supplemental material has been provided by the authors to give readers additional information about their work.

© 2023 Shapiro DJ et al. *JAMA Network Open.*

eAppendix. Administrative Codes

Variable	Type of Code[1]	Definition
**Albuterol**	NDC, CPT, HCPCS	Prescriptions: Albuterol, levalbuterol<br><br>Treatment: J7607, J7609, J7610, J7611, J7612, J7613, J7614, J7615, J7620, 94640, 94644, 94645
**Corticosteroids**	NDC, HCPCS	Prescriptions: Dexamethasone, prednisone, prednisolone, methylprednisolone<br><br>Treatment: J7506, J7509, J7510, J8540, J1020, J1030, J1040, J1720, J2920, J2930, J3303, J0702, J3301, J3300, J3302, J1100, J1094, J2650, J1700, J1710 J2650, J7512, J1700, J1710, J3304, Q9993, C9469
**Antibiotics**	NDC	Prescriptions: Amoxicillin, amoxicillin/clavulanate, penicillin, cephalexin, cefadroxil, cefuroxime, cefpodoxime, cefixime, cefdinir, clindamycin, azithromycin, levofloxacin, trimethoprim/sulfamethoxazole
**Intravenous fluids**	CPT, HCPCS	96360, 96361, S5010, J7030, J7040, J7042, J7050, J7120, J7121
**Chest radiography**	CPT	71010, 71015, 71020, 71021, 71022, 71023, 71030, 71034, 71035, 71045, 71046, 71047, 71048
**Viral testing**	CPT	87807, 87280, 87420, 87631, 87632, 87633, 87798, 87636, 87637, 87426, 87811, U0001, U0002, U0003, U0004, U0005, 87426, 87635, 87811, 87275, 87276, 87279, 87280, 87400, 87420, 87501, 87502, 87503, 87631, 87634, 87804, 87807, 87428, 87636, 87637, 0240U, 0241U
**Office visits[2]**	CPT, HCPCS	T1015, 99201-99205, 99211-99215, 99381-99385, 99391-99395

1. CPT: Current Procedural Terminology; HCPCS: Healthcare Common Procedure Coding System; NDC: National Drug Code Directory; ICD-10: International Classification of Diseases, 10th Revision

2. Excludes visits to medical/pediatric/surgical subspecialists, optometry, dentistry, pharmacy, and physical therapy.

© 2023 Shapiro DJ et al. *JAMA Network Open.*
