# Supplementary material for: National Patterns of Outpatient Follow-Up Visits After Emergency Care for Acute Bronchiolitis
Source: JAMA Netw Open. 2023 Oct 27;6(10):e2340082. doi: 10.1001/jamanetworkopen.2023.40082 (PMC10611989; doi:10.1001/jamanetworkopen.2023.40082)
Supplement: Supplement 2. — Data Sharing Statement [file jamanetwopen-e2340082-s002.pdf]

Data Sharing Statement

Shapiro. National Patterns of Outpatient Follow-Up Visits After Emergency Care for Acute Bronchiolitis. *JAMA Netw Open*. Published October 27, 2023.
doi:10.1001/jamanetworkopen.2023.40082

Data
Data available: No
